# Immune checkpoint inhibitor-related pneumonitis in non-small cell lung cancer: A review

**DOI:** 10.3389/fonc.2022.911906

**Published:** 2022-08-16

**Authors:** Yuxuan Hao, Xiaoye Zhang, Li Yu

**Affiliations:** Department of Oncology, Shengjing Hospital of China Medical University, Shenyang, China

**Keywords:** immune checkpoint inhibitor, immune checkpoint inhibitor-related pneumonitis, immune-related adverse events, immunotherapy, non-small cell lung cancer

## Abstract

Immune checkpoint inhibitors (ICIs) have shown definite therapeutic effects in various types of cancers, especially non-small cell lung cancer (NSCLC). However, ICIs have unique side effects, called immune-related adverse events (irAEs), which can occur in various systems throughout the body. Among such irAEs, immune checkpoint inhibitor-related pneumonitis (ICI-P) is a fatal adverse reaction. In this review, we discussed the risk factors, pathogenesis, clinical characteristics, radiological manifestations, pathological features, diagnosis, grading, and management of ICI-P in NSCLC and the relationship between ICI-P and the efficacy of ICI therapy. In addition, we discussed the predictive factors for ICI-P. This review will play a crucial role in the prediction, evaluation, and management of ICI-P for widespread application of immunotherapy.

## Introduction

Lung cancer is one of the most common malignant tumours worldwide, with non-small cell lung cancer (NSCLC) accounting for 85% of cases (1, 2). According to the recent population-based cancer incidence and mortality data reported by the American Cancer Society, which is compiled every year, the incidence of cancer is gradually decreasing, and the decline in the number of lung cancer cases is particularly pronounced (3). In addition, the mortality rate of lung cancer has declined significantly, which is related to improved management.

Immune checkpoint inhibitors (ICIs) exert significant clinical therapeutic effects and have accelerated the treatment of advanced cancer in the new era of immunotherapy. The use of ICIs has shown great success in improving the overall survival (OS) and progression-free survival (PFS) rates of NSCLC (4–7). ICIs have been approved as a first-line treatment for advanced driver gene-negative NSCLC owing to their superior efficacy and no evident side effects compared with conventional chemotherapy (8). However, they can result in systemic reactions called immune-related adverse events (irAEs) (9) that are completely different from adverse reactions resulting from conventional chemotherapy.

IrAEs can affect all organs of the body, including the skin, gastrointestinal tract, liver, kidneys, lungs, endocrine organs, and the central nervous system (9–11). Among irAEs, immune checkpoint inhibitor-related pneumonitis (ICI-P) is a rare but fatal reaction (12, 13). ICI-P is defined as the development of dyspnoea and/or other respiratory symptoms and the appearance of a new infiltrative shadow on chest imaging after the patient has been treated with ICI, except for clinical conditions such as lung infection or tumour progression. According to the data of clinical trials, the incidence of ICI-P is 3%—6.3% in NSCLC, and the mortality rate is <1% (14–16). However, in previous epidemiological studies, the incidence of ICI-P varied greatly, ranging from 2.7% to 19% in NSCLC (17–20) (**Table 1**). Patients with lung cancer are more likely to develop ICI-P than patients with other types of cancer (17). The onset of ICI-P is earlier in patients with lung cancer (78 days) than in patients with non-lung cancer (186 days) (21). Recent real-world statistical data show that in clinical practice, the incidence of ICI-P is higher than that reported in pivotal trials, leading to the approval of programmed death-(ligand) 1 (PD-[L]1) inhibitors (22) by the United States Food and Drug Administration. High-grade ICI-P is extremely dangerous and often threatens the lives of patients. Therefore, clinicians should make rapid and accurate decisions for providing reasonable and effective treatment for ICI-P.

**Table 1:** Published meta-analysis and clinical trials on immune checkpoint inhibitor-related pneumonitis.

Author	Year	Numbers of patients/trials	ICI type	Tumour type	Incidence (%)	Mortality(%)	Grade≥3 (%)
Khunger M(14)	2017	5038/19	Anti-PD-1 Anti-PD-L1	NSCLC	PD-1: 3.6 PD-L1: 1.3	N/A	PD-1: 1.1 PD-L1: 0.4
DeVelasco G(15)	2017	11454/21	Anti-PD-1 Anti-PD-L1 Anti-CTLA-4	NSCLC,SCLC,Melanoma,etc.	All patients: 2.6	<1	All patients: 1.1
Nishino M(17)	2016	4496/20	Anti-PD-1 Anti-CTLA-4	NSCLC,Melanoma,RCC	All patients: 2.7 NSCLC: 4.1 Melanoma: 1.6 Monotherapy: 1.6 Combined therapy: 6.6	NSCLC: 0.4	All patients: 0.8 NSCLC: 1.8 Melanoma: 0.2 Monotherapy: 0.2 Combined therapy: 1.5
Cho JY(18)	2018	167/1	Anti-PD-1 Anti-PD-L1	NSCLC	All patients: 13.2 Monotherapy: 13.6 Combined therapy: 10	All patients: 18.2	All patients: 4.2 Monotherapy: 4.1 Combined therapy: 5
Suresh K(19)	2018	205/1	Anti-PD-1 Anti-PD-L1	NSCLC	All patients: 19.02	N/A	All patients: 11.7
Ono K(20)	2021	203/1	Anti-PD-1	NSCLC	All patients: 13.79	N/A	All patients: 3.44

ICI, immune checkpoint inhibitor; NSCLC, non–small cell lung cancer; SCLC, small cell lung cancer; RCC, renal cell carcinoma; PD-1, programmed cell death-1; PD-L1, programmed cell death-ligand 1inhibitor; CTLA-4, cytotoxic T lymphocyte associated protein 4; N/A, not applicable.

## Risk factors for ICI-P

Many potential factors can increase the incidence of ICI-P in NSCLC patients treated with ICIs. Among the 97 patients who participated in the subgroup analysis of the Keynote-001 trial, OS was significantly longer after pembrolizumab administration in patients who had previously received radiotherapy than in patients who had not received radiotherapy. However, 3 (3/24, 13%) patients who underwent thoracic radiotherapy had developed ICI-P, whereas only 1 (1/72, 1%) patient who did not receive thoracic radiotherapy had developed ICI-P. Therefore, the probability of treatment-related pulmonary toxicity is higher in patients who have received radiotherapy than in patients who have not received radiotherapy (23). Other clinical studies have reported that patients who have received thoracic radiotherapy are more likely to have ICI-P (24–26) and respiratory failure (24). According to several retrospective studies, pre-existing history of interstitial pneumonitis is also associated with the incidence of ICI-P (27, 28). The development of ICI-P is independently associated with the presence of baseline fibrosis on computed tomography (CT) of the chest, which is a composite measure of obstructive lung disease (29). Cho et al. reported the same phenomenon, indicating that a pre-existing pulmonary disease associated with a significantly higher incidence of ICI-P, which could explain why ICI-P is more common in lung cancer patients than in other cancer types (18).

According to a recent meta-analysis of ICI clinical trials, the total incidence of ICI-P after single and combination therapy is 1.6% and 6.6%, respectively, suggesting that the risk of ICI-P is higher after combination therapy than after single therapy (17). In another study, a higher incidence of ICI-P was seen with the risk ratio of all grades of ICI-P increasing up to 2.92 after combination therapy (30). According to a meta-analysis, ICI-P was the most common cause of anti-PD-1/PD-L1-related fatalities (35%). In addition, toxicity-related fatality rates were higher in patients who received combination therapy of PD-1/PD-L1 plus CTLA-4 (1.23%) than in those who received single therapy with anti-PD-1 (0.36%), anti-PD-L1 (0.38%), or anti-CTLA-4 (1.08%) (31). The incidence of ICI-P was higher in patients treated with sequential therapy with targeted agents (18.8%) within 8 weeks of ICI treatment than in patients treated with cytotoxic agents (7.4%) and in patients not treated with chemotherapy (5.1%). The onset of ICI-P was earlier in patients who received sequential therapy with targeted agents after immunotherapy than in those who received sequential therapy with cytotoxic drugs (35 days versus 62 days, respectively). Patients who received targeted agents within 8 weeks of immunotherapy had a higher chance (100%) of developing ≥grade 3 ICI-P than those treated with cytotoxic agents (0%). Among 23 patients with ICI-P, 16 patients (69.6%) required intravenous steroids. Despite receiving high-dose systemic intravenous steroids, 1 patient with grade 4 pneumonitis recovered, whereas 6 (26.1%) patients died (32). Some findings showed that PD-1 inhibitors were associated with a higher incidence of ICI-P compared with PD-L1 inhibitors (immune monotherapy) (33–35). In a study by Khunger, compared with PD-L1 inhibitors (1.3%), PD-1 inhibitors were associated with a higher risk of ICI-P (3.6%). In addition, the incidence of >grade3 ICI-P was higher in patients receiving anti-PD-1 therapy (1.1%) than in those receiving PD-L1 therapy (0.4%) (14).

A retrospective study enrolling 1826 patients with cancer reported that ICI-P occurred more frequently in men and former or current smokers (64 [3.5%] patients) (36). Nakahama reported that tumour invasion in the central airway (TICA) was associated with an increased risk of ICI-P. Patients with TICA had a higher risk of ICI-P than patients with a history of radiotherapy, which is a well-known risk factor for ICI-P (37). Based on the conclusions of these two studies, the incidence of ICI-P is higher in NSCLC (especially squamous cell lung cancer) than in melanoma (38, 39). Furthermore, a study involving 837 patients showed that 354 (42.3%) patients aged ≥ 65 years had a significantly increased risk of developing ICI-P, compared with 483 (57.7%) patients aged < 65 years, with a risk ratio of 2.12 (40).

In conclusion, patients with the following characteristics: male, former or current smoker, ≥65 years old, previous chest radiotherapy, previous lung disease, combination therapy, and TICA are predisposed to ICI-P after immunotherapy (**Table 2**). Clinicians should be cautious while using ICIs to treat the aforementioned susceptible populations and keep patients under careful observation after ICI therapy.

**Table 2:** Risk factors of immune checkpoint inhibitor-related pneumonitis.

**Patient characteristics:**
Λ male
Λ ≥ 65 years old
Λ former or current smoker
Λ previous lung disease
**Tumour:**
Λ lung cancer > other cancer types
squamous cell lung cancer > non-squamous cell lung cancer
Λ tumour invading the central airway
**Treatment:**
Λ previous chest radiotherapy
Λ Anti-PD-1 > Anti-PD-L1
Λ combination therapy
ICI and targeted drug > ICI and cytotoxic drug> single ICI
Anti-PD-1/PD-L1 and Anti-CTLA-4 > immune monotherapy

PD-1, programmed cell death-1; PD-L1, programmed cell death-ligand 1 inhibitor; CTLA-4, cytotoxic T lymphocyte associated protein 4; ICI, immune checkpoint inhibitor; NSCLC, non–small cell lung cancer; SCLC, small cell lung cancer; CT, computed tomography.

## Pathogenesis of ICI-P

The mechanisms of ICI-P remain unclear; however, some theories based on the mechanism of action of ICIs and related studies are described below. Activated T cells, B cells, NK T cells and myeloid cells express PD1 (41). Tumour cells express PD-L1, which is upregulated in macrophages, dendritic cells, fibroblasts and activated T cells (42). The interaction between PD-L1 and PD1 inhibits the function, differentiation and survival of T cell (41). Anti-PD(L)-1 improves the anti-tumour immune response by activating T cells and relieving the inhibition of associated signalling pathways. CTLA-4 is an inhibitory receptor belonging to the CD28 immunoglobulin subfamily, expressed mainly by T-cells, which inhibits binding of CD28 on T cells, to B7 proteins on antigen-presenting cells (APCs), thereby impairing the costimulatory effect of T cells the costimulation on T cells (43, 44). In addition, CTLA4 can also interferes with Treg cell function (45). In a study on a knockout mouse model, mice lacking the CTLA-4 gene died of lymphoproliferation, whereas those lacking PD-1 developed lupus-like autoimmune diseases (46, 47). The occurrence of ICI-P may be associated with excessive T cell activation and tumour microenvironment disturbance. Although ICIs promote lymphocyte activation against tumours, activated T cells can damage alveolar cells, leading to ICI-P (48, 49). A study by Suresh reported a marked increase in the number of lymphocytes, especially CD4+ T cells, in the bronchoalveolar lavage (BAL) of patients with ICI-P (50). The tumour microenvironment includes both immune cells and associated cytokines. Disturbance in the tumour microenvironment owing to ICI use may also contribute to the development of ICI-P. A study by Catacchio highlighted the significance of the tumour microenvironment (44). ICIs are immunotherapeutic agents with a specific target. Off-target toxicity is a specific mechanism by which targeted therapy causes negative effects (51). CD8+ cytotoxic T lymphocyte-mediated cell lysis induces the release of neoantigens, tumour antigens, and autoantigens from normal tissues. The immune tolerance of normal tissues is reduced as a result of this phenomenon known as “epitope spreading,”, which may lead to the development of ICI-P (52).

## Clinical characteristics

ICI-P is a unique toxic reaction that occurs after immunotherapy. Although it is relatively rare, it is one of the most important causes of death caused by ICIs in patients with NSCLC. A meta-analysis by Mizuki Nishino (17) showed that the overall incidence of ICI-P after PD-1 inhibitor monotherapy was 2.7% for all-grades of ICI-P and 0.8% for ≥grade 3 ICI-P. However, the incidence was higher among patients with NSCLC than with melanoma and kidney cancer, for all-grade (4.1%) and ≥grade 3 ICI-P (1.8%). The extent of involvement of the lungs in ICI-P is highest in the lower lungs, followed by the middle and upper lungs. In a clinical study by Myriam Delaunay (53), the average time required for the onset of ICI-P after introducing immunotherapy was 2.3 (0.2-27.4) months. A majority (42.2%) of patients developed ICI-P within < 2 months of introducing immunotherapy; the time to development of ICI-P was 2-4 months in 26.6%, 4-6 months in 17.2% and > 6 months in 14.1% of patients. Another study also showed that the onset of all grades of ICI-P was early, usually within 6 months of initiating immunotherapy, with higher-grade ICI-P occurring earlier than lower-grade ICI-P (19).

ICI-P is non-infectious pneumonitis, and its clinical manifestations are different from ordinary pneumonitis (54). A study by Myriam Delaunay showed that the most common symptoms of ICI-P were dyspnoea (80.3%) and cough (52.5%). Fever (32.8%) and asymptomatic conditions (6.6%) were less common (53). Another clinical study by Tomomi W Nobashi reported that one of the major symptoms of ICI-P was fever, lasting for a few days or more. They found that the symptoms with a higher incidence were fever (30%), dyspnoea (26%), low oxygen saturation (15%) and cough (15%), whereas those with a relatively low incidence were malaise (7%), rash (4%) and anorexia (4%). In addition, thyroid dysfunction and rashes were common in patients with and without ICI-P; however, the frequency of incidence was significantly higher in patients with ICI-P (21).

### Radiological manifestations

When ICI-P is suspected, clinicians should make accurate and rapid decisions, because ICI-P has characteristics that demand urgency in treatment. However, the clinical manifestations of ICI-P are diverse, and it is difficult to predict the occurrence of ICI-P before initiating treatment. CT of the chest plays a significant role in the diagnosis of ICI-P. Understanding the features of CT of the chest in ICI-P is important for prompt treatment. At present, the imaging classifications are mainly divided into the following categories: organising pneumonitis (OP), non-specific interstitial pneumonitis (NSIP), hypersensitivity pneumonitis (HP) and diffuse alveolar damage (DAD). DAD is also called acute respiratory distress syndrome (ARDS). The severity of these conditions is graded as follows: DAD>NSIP/HP>OP. In terms of incidence rate, OP has the highest incidence (65%), NSIP has a lower incidence (15%) and HP and DAD have the lowest incidence (10%) (55, 56). The radiological features of cryptogenic organising pneumonitis (COP) may be a sign of enhanced efficacy of ICIs (57). In addition, signs of ground-glass opacity (GGO), consolidations, traction bronchiectasis, nodular lesions, and reversed halo can be observed on CT. Among the five major types of signs, GGO is observed in a majority of patients, followed by consolidations. Although the reversed halo sign is rare, it is a typical finding in OP (18).

## Pathological features

Pathological methods are becoming increasingly essential for the diagnosis of ICI-P. They can be used to rule out infectious pneumonitis and tumour progression. However, BAL and lung biopsy are not routinely performed in patients with ICI-P. In a retrospective study by Brandon T on 9 patients with ICI-P (58), OP was the most common histological pattern (7 patients). Among the 9 patients, 3 had concomitant ambiguous non-necrotising granulomas in the airway, and 2 presented with more acute symptoms, with histological changes indicating severe acute lung injury. In addition, 1 patient showed a pattern of acute fibrinous pneumonitis, and 1 patient with acute respiratory failure showed a pattern of acute and organising DAD. All 9 patients showed patchy accumulation of foamy macrophages in the airway and vacuolisation of type II pneumocytes. BAL has been used in a few studies on patients with NSCLC with ICI-P. Sabino Strippoli (59) analysed the characteristics of BAL in patients with melanoma with ICI-P and showed that cellular analysis using BAL revealed typical and homogeneous features with increased lymphoid population, relevant enrichment of CD8 + T cells and consequent inversion of the CD4/CD8 ratio. Moreover, the proportion of activated CD3 + HLA-DR + T cells was associated with the grading of adverse events. It has been reported that a major feature of BAL analysis in ICI-related NSCLC is an increase in the proportion of lymphocytes (60). The proportion of BAL lymphocytes, mainly CD4 + T cells, increases in ICI-P. An increase in the number of BAL central memory T (Tcm) cells, evidence of type I polarisation, and decreased expression of CTLA-4 and PD-1 in BAL Tregs indicate both activation of pro-inflammatory subpopulations and a weakened inhibitory phenotype. In a study, the myeloid immune population in BAL supernatants in ICI-P showed increased expression of IL-1β and decreased counter-regulation of IL-1RA expression, with increased levels of Tcm chemoattractants. These dysregulated immune cell subsets may represent possible targets for the treatment of pathological irAEs (50). Bronchoscopy plays an important role in the diagnosis of acute lung injury and fibrosis (61).

## Diagnosis

The diagnosis of ICI-P requires a comprehensive consideration of the clinical symptoms, as well as general bloodwork, CT imaging, and invasive evaluation (BAL or lung biopsy). Exclusion diagnosis is also an important strategy. Infectious pneumonitis, radiation pneumonitis (RP), tumour progression, carcinomatous lymphangitis, and pulmonary oedema caused by heart failure or myocarditis are common differential diagnoses. Establishing a diagnosis of ICI-P requires the exclusion of diseases mentioned in **Table 3** (62–64). Among them, the presentation of RP and ICI-P is similar to that of interstitial pneumonitis. Therefore, it is difficult to distinguish clinically RP from ICI-P in patients who have undergone both radiotherapy to the chest and immunotherapy. However, there are some differences in terms of CT location distribution between the two types of pneumonitis. On CT of the chest, RP usually shows sharp margins; thin, dense plaques or streak-like changes in the lung ipsilateral to a lesion consistent with the extent of irradiation and to a lesion that is not consistent with the normal lung tissue structure (not distributed based on lung field or lung segment) (65). However, ICI-P is usually bilateral, involves multiple lung lobes and shows no sharp borders on CT, and the ICI-P area usually does not cross the lung fissures (66).

**Table 3:** Differential diagnosis of immune checkpoint inhibitor-related pneumonitis.

Differential diagnosis	Description
Infectious pneumonitis	• Most patients have symptoms of fever and expectoration, and antibiotic treatment is effective. • Elevation of serum inflammatory response indicators (including WBC, CRP, PCT, IL-6, etc.). • Positive results of pathogen detection (including nasal swab, sputum culture, blood culture and BAL). • CT findings: consolidation, air bronchogram sign, silhouette sign, tree-in-bud sign, etc. Usually distributed by lung fields or segments.
Radiation pneumonitis	• Typically develops 4 to 12 weeks after completing radiotherapy. • CT findings: patchy lesions, diffuse ground-glass opacity, traction bronchiectasis and scar-like lesions in the irradiated field.
Tumour progression or carcinomatous lymphangitis	• Metastasizes in the lungs or grows and spreads along the lymphatic vessels. • CT findings: new nodules, ground-glass opacities, reticular nodules, thickened bronchial bundles and beaded thickening of interlobular septa. • Exfoliative cytology, BAL and lung biopsy will play an important role in the diagnosis.
Pulmonary oedema due to heart failure or myocarditis	• Specific clinical manifestations: paroxysmal nocturnal dyspnea, dyspnea after exercise, pink foam sputum, etc. • CT findings: interlobular septums, fissures, peribronchovascular interstitium thickening, cardiomegaly, pleural effusion, Kerley B lines and increased artery to bronchus ratio.

WBC, white blood cell; CRP, C-reactive protein; PCT, procalcitonin; IL-6, interleukin-6; BAL, bronchoalveolar lavage; CT, computed tomography.

## Grading and management of ICI-P

### Grading

According to the latest National Comprehensive Cancer Network (NCCN) guidelines, ICI-P is divided into three levels and four grades as follows: mild (G1), moderate (G2) and severe (G3-4) (**Table 4**). Grade G1 refers to asymptomatic disease; ICI-P is confined to one lobe or < 25% of the lung parenchyma. Grade G2 marks the appearance of new symptoms such as shortness of breath, cough, chest pain, fever, and hypoxia; with the involvement of multiple lung lobes, affecting 25%-50% of the lung parenchyma; It affects daily life and requires drug intervention. Grade G3 refers to the appearance of serious new symptoms. It involves all lung lobes or > 50% of the lung parenchyma. Patients with G3 ICI-P have limited self-care ability and require oxygen supplementation. Grade G4 refers to life-threatening respiratory system damage, such as acute respiratory distressyndrome (ARDS), which requires emergency care (67).

**Table 4:** Grading of immune checkpoint inhibitor-related pneumonitis.

Level	Grade	Description
Mild	G1	Λ asymptomatic
		Λ ICI-P is confined to one lobe or < 25% of the lung parenchyma
Moderate	G2	Λ the appearance of new symptoms such as shortness of breath, cough, chest pain, fever, and hypoxia
		Λ multiple lung lobes are involved, affecting 25%—50% of the lung parenchyma
Severe	G3	Λ the appearance of serious new symptoms
		Λ involves all lung lobes or > 50% of the lung parenchyma
	G4	Λ life-threatening respiratory system damage
		Λ ARDS

ICI-P, immune checkpoint inhibitor-related pneumonitis; ARDS, acute respiratory distress syndrome.

### Management

Steroid therapy is a routine strategy for the management of ICI-P. Regular and sufficient use of steroids can help to treat 70—80% of patients with ICI-P (16). According to the current consensus on ICI-P treatment, steroid therapy should be initiated after a confirmed diagnosis of ≥G2 ICI-P. Patients with G1 ICI-P can be temporarily observed, and the use of ICIs should be suspended (1-2 weeks). However, if there are signs of progress of ICI-P, steroid therapy should be initiated. For patients with G2 ICI-P, prednisone/methylprednisolone at a dose of 1—2 mg/kg/day (Treatment until symptoms improve to ≤ grade 1, then taper over 4-6 weeks) is usually administered. For patients with G3-4, methylprednisolone at a dose of 1—2 mg/kg/day (taper over ≥6 weeks) is usually administered (67). Some patients are not sensitive to steroid therapy (no improvement after 48 hours for G3-4 ICI-P). This condition is usually called steroid-refractory pneumonitis (68), the criterion of which is mainly based on clinical symptoms and chest CT. In this case, the following treatments can be considered: 1) Intravenous administration of infliximab at a dose of 5 mg/kg, which can be repeated after 14 days at the discretion of a physician; 2) Intravenous injection of immunoglobulin; 3) Mycophenolate mofetil at a dose of 1-1.5 g twice daily (BID); the dosage can be gradually decreased over time (67, 69, 70). During treatment, the efficacy should be continuously monitored. If the infection has not been completely ruled out, empirical use of antibiotics should be considered. In terms of supportive treatment, clinicians should provide corresponding respiratory and systemic support to patients and actively deal with their complications. For reinitiating ICI treatment, a cohort study showed that after re-challenge with the same ICI, the recurrence rate of the same irAE associated with the discontinuation of ICI therapy was 28.8%. In such cases, clinicians can consider resuming ICI treatment for selected patients, who should be monitored appropriately (71, 72).

## Discussion

### Association between the occurrence of ICI-P and the outcome of cancer treatment

Some scholars have compared a large amount of research data and concluded that the occurrence of irAEs is directly proportional to the prognosis (73). The occurrence of irAEs indicates that immunotherapy has activated the immune system of patients. Patients with greater toxicity to immune drugs can attain better curative effects, leading to prolonged PFS and OS (20). In a meta-analysis (74), irAEs, especially endocrine, dermatologic and low-grade irAEs, were significantly associated with better ICI outcomes in patients with cancer. In addition, the development of irAEs was associated with the beneficial effects of treatment on survival in patients with cancer treated with PD-1 inhibitors but not in those treated with CTLA-4 inhibitors. Patients receiving immune monotherapy have more significant benefits than patients receiving combination therapy. Syed Hussaini reported a similar conclusion (75). In patients treated with ICIs, the occurrence of irAEs is positively correlated with objective response rate (ORR), PFS and OS but is not associated with the site of disease, type of ICIs and irAEs. Patients with ≥grade 3 ICI-P have better ORR but worse OS. However, some studies have reported more positive results, indicating that the OS of patients with multiple irAEs is significantly better than that of patients with a single irAE (76). Although studies have shown that endocrine, skin, and low-grade irAEs are associated with the efficacy of immunotherapy, some studies have reported that ICI-P can significantly improve recurrence-free survival (RFS) (77). Studies have also shown that the ORR and PFS of patients with ICI-P are significantly better than those of patients with irAE-non-ICI-P and non-irAEs (27). In a study by Shankar B, the PFS and OS of the ICI-P group were better than those of the non-ICI-P group (76). The incidence of low-level ICI-P can prolong PFS and OS, and increase ORR. High-grade ICI-P does not benefit OS but can help in achieving better ORR (75) (**Table 5**)

**Table 5:** Published researches on association between the occurrence of ICI-P and the outcome of immunotherapy in NSCLC.

Author	Year	Numbers of patients/trials	ICI type	Incidence of ICI-P (%)	ORR(%)	PFS(months)	OS(months)
Ono K (20)	2021	203/1	Anti-PD-1	14	ICI-P: 68 Non-ICI-P: 20	ICI-P: 18.9 Non-ICI-P: 3.9	ICI-P: 27.4 Non-ICI-P: 14.8
Sugano T (27)	2020	130/1	Anti-PD-1 Anti-PD-L1	12	ICI-P: 63 Other irAEs: 43 Non-irAEs: 22	ICI-P: 15.9 Other irAEs: 5.4 Non-irAEs: 3.3	N/A
Haratani K (73)	2018	134/1	Anti-PD-1	4	N/A	IrAEs: 9.2 Non-irAEs: 4.8	IrAEs: NR Non-irAEs: 11.1
Shankar B (76)	2020	623/1	N/A	Monotherapy: 12 Combined therapy: 9	N/A	Single irAE: 10.9 Multisystem irAEs: 5.1 Non-irAEs: 2.8	Single irAE: 21.8 Multisystem irAEs: 12.3 Non-irAEs: 8.7
Hussaini S (75)	2021	2859/19	Anti-PD-1 Anti-PD-L1	N/A	IrAEs: 41.49 Non-irAEs: 18.01	IrAEs: 8.97 Non-irAEs: 3.06	IrAEs: 19.07 Non-irAEs: 7.45

NSCLC, non–small cell lung cancer; PD-1, programmed cell death-1; PD-L1, programmed cell death-ligand 1inhibitor; CTLA-4, cytotoxic T lymphocyte associated protein 4; ICI, immune checkpoint inhibitor; irAEs, immune-related adverse events; NR, not reached; OS, overall survival; PFS, progression free survival; ORR, bjective response rate; N/A, not applicable.

### Markers predicting the occurrence of ICI-P

The incidence of serious ICI-P after immunotherapy adversely affects the survival and quality of life of patients. The discovery and optimisation of predictive factors through laboratory tests can help clinicians to predict the occurrence of ICI-P, discontinue the use of ICIs in time or administer steroids early and may help in prolonging the survival of patients. A study showed that the expression of PD-L2 may be related to the incidence of irAEs in patients with NSCLC treated with PD-1 inhibitors. In addition, pre-existing autoimmunity markers such as the rheumatoid factor have been identified as independent predictors of skin reactions caused by ICIs (78). Thyroid dysfunction is more common in patients with anti-thyroid antibodies (79). However, to the best of our knowledge, studies have not reported the specific predictors of ICI-P. Therefore, relevant fundamental research is warranted to guide the application of clinical immunotherapy.

## Conclusion

Clinicians should be cognizant of adverse reactions caused by ICIs, especially ICI-P. It is important to make an assessment before administering medications and pay attention to high-risk groups. After the administration of immunotherapeutic drugs, clinicians should pay close attention to changes in the condition of patients. Based on the combination of clinical manifestations, imaging data, and pathological characteristics of patients, ICI-P can be easily diagnosed, considering that infectious pneumonitis is excluded. ICI-P is mild in most cases and can be cured by appropriate treatment such as discontinuing immunotherapy or using steroids. If severe ICI-P occurs, immunotherapy should be promptly discontinued, and steroids and immunosuppressive therapy should be administered. Early intervention has a great impact on the survival and quality of life of patients. More studies are required for steroid-refractory pneumonitis, because, at present, an effective standard treatment plan is not available. In addition, further investigation is warranted to identify the predictors of ICI-P in the future.

## Author contributions

All authors planned and wrote the manuscript and contributed to the article and approved of the submitted version.

## Conflict of interest

The authors declare that the research was conducted in the absence of any commercial or financial relationships that could be construed as a potential conflict of interest.

## Publisher’s note

All claims expressed in this article are solely those of the authors and do not necessarily represent those of their affiliated organizations, or those of the publisher, the editors and the reviewers. Any product that may be evaluated in this article, or claim that may be made by its manufacturer, is not guaranteed or endorsed by the publisher.
